# Presence of dengue virus RNA in urine and oral fluid of laboratory-confirmed dengue patients: Implications for wastewater surveillance

**DOI:** 10.1016/j.bjid.2024.104484

**Published:** 2024-11-29

**Authors:** Christine Stauber, Leile Camila Jacob-Nascimento, Caroline Grosch, Moisés da Silva Sousa, Moyra M. Portilho, Rosângela O. Anjos, Margo A. Brinton, Uriel Kitron, Mitermayer G. Reis, Guilherme S. Ribeiro

**Affiliations:** a Georgia State University, School of Public Health, Atlanta, USA; b Fundação Oswaldo Cruz, Instituto Gonçalo Moniz, Salvador, BA, Brazil; c Universidade Federal da Bahia, Faculdade de Medicina da Bahia, Salvador, BA, Brazil; d Georgia State University, College of Arts and Sciences, Department of Biology, Atlanta, USA; e Emory University, Department of Environmental Sciences, Atlanta, USA; f Yale University, Yale School of Public Health, New Haven, USA

**Keywords:** Epidemiology, Dengue virus, Wastewater-based epidemiology

## Abstract

**Introduction:**

Dengue cases in the Americas in 2024 have reached record highs, especially in Brazil. However, surveillance remains suboptimal and new methods are needed to monitor Dengue Virus (DENV) spread. To assess whether wastewater-based epidemiology would be a useful tool, we investigated the presence of DENV RNA in dengue patients’ urine and oral fluid from an endemic area to inform how shedding in these fluids occurs and provide insight for wastewater surveillance.

**Methods:**

We examined how often DENV RNA is detected in urine and oral fluid from dengue patients confirmed by serum RT-qPCR, NS1 ELISA or IgM seroconversion in Salvador, Brazil.

**Results:**

Of 88 confirmed cases, 9.1 % were positive for DENV RNA in urine (7/88) or oral fluid (1/88). Of 53 serum RT-qPCR-positive patients, 6 (11.3 %) showed detectable DENV RNA in acute- or convalescent-phase urine. Patients with RT-qPCR-positive urine had a lower frequency of DENV IgG in acute-phase serum (a proxy for secondary infection) (57 % vs. 74 %) and a lower median serum RT-qPCR cycle threshold than those with negative urine (21.8 vs. 23.9).

**Conclusion:**

The low presence of DENV RNA in urine suggests that additional research is needed to evaluate whether using wastewater-based epidemiology to monitor DENV transmission is possible.

## Highlights

• Patients with dengue can shed dengue virus RNA in urine and oral fluids.
• Dengue RNA was detectable in acute and convalescent urine samples.
• Wastewater-based epidemiology may be useful for monitoring dengue transmission.

In the Americas, Dengue Virus (DENV) has the highest incidence of all mosquito-borne diseases, with cyclic epidemics occurring every 3 to 5 years.^1^ In 2024, the number of dengue cases sharply increased, surpassing previous records, especially in Brazil.^1^ However, surveillance remains suboptimal because most DENV infections are asymptomatic or, when symptomatic, are not reported.^2^ Furthermore, serological surveys to determine population exposure levels have reduced accuracy since the emergence of the Zika Virus (ZIKV) due to antibody cross-reactivity between orthoflaviviruses.^3^ Therefore, alternative methods to assess the risk of infection and transmission are greatly needed.

Wastewater-Based Epidemiology (WBE) does not require detecting and reporting suspected cases during medical care or invasive sampling techniques. It provides a snapshot of community health and has proven successful during the COVID-19 pandemic.^4^ Several arboviruses have been detected in urine samples, and DENV has recently been identified in wastewater.^5^ While DENV transmission does not occur through direct contact with body fluids containing the virus, understanding the potential for DENV RNA shedding from biological samples into wastewater may help determine whether WBE can be an effective surveillance tool.

We investigated the presence of DENV RNA in paired urine and oral fluid from patients with laboratory-confirmed dengue in Salvador, Brazil, in hopes of contributing insights about the feasibility of using WBE to monitor DENV transmission.

Since 2016, a surveillance study to detect cases of symptomatic arbovirus infection among patients seeking care at a public health clinic in Salvador, Brazil, has been ongoing.^2^ The inclusion criteria for this study were residence in Salvador, age ≥ 6 months, and having a reported or measured fever (temperature ≥37.8 °C; 100.04°F) or skin rash within the seven days before enrollment. All participants and legal guardians of children < 18 years old provided written informed consent; those aged 5 to 17 also provided written assent. The study was approved by the Oswaldo Cruz Foundation (CAAE 55,904,616.4.0000.0040).

Clinical and epidemiological data and biological samples were collected at enrollment (acute-phase blood, oral fluid, and urine) and at the convalescent phase of illness (blood and urine collected 10‒45 days post the acute-phase samples). Serum samples obtained by blood centrifugation were stored at -20 °C until serological testing. Additionally, serum, urine, and oral fluid samples were stored at -80 °C until molecular testing.

Acute-phase sera underwent RNA extraction using Maxwell® 16 Viral Total Nucleic Acid Purification Kit (Promega, Madison, WI, USA), QIAamp Viral RNA Mini kit (QIAGEN, Hilden, Germany), or Quick-DNA/RNA Viral MagBead R2140 (Zymo Research, Tustin, CA, USA) with the KingFisher Sample Purification Systems (Thermo Fisher Scientific, Waltham, MA, EUA). The extracted RNA was then analyzed with RT-qPCR for DENV, ZIKV and chikungunya virus using the CDC TRIOPLEX RT-qPCR.^6^ DENV-positive serum underwent molecular typing as previously described.^7^

Acute- and convalescent-phase sera were tested by DENV IgM Enzyme-Linked Immunosorbent Assay (ELISA) (Panbio Diagnostics, Brisbane, Australia). Acute-phase sera were tested by DENV Non-Structural-1 (NS1) antigen (Panbio Diagnostics, Brisbane, Australia) and DENV IgG ELISAs (Panbio Diagnostics, Brisbane, Australia). The last test was used only for the laboratory-confirmed dengue cases to differentiate primary and secondary infections, as defined by a negative and a positive result, respectively. Acute DENV infection was determined by a positive DENV result by RT-qPCR or NS1 ELISA or by detecting DENV IgM seroconversion between paired serum samples.

DENV RNA was analyzed in urine and oral fluid samples using the same procedures for RNA extraction and RT-qPCR for sera.^6^ Oral fluid samples were processed before RNA extraction as previously described.^8^

Between September 2016 and May 2023, the study enrolled 3364 patients and dengue was confirmed in 119 (3.5 %). Of these, 88 (73.9 %) had acute-phase oral fluid and urine collected and 53 (60.2 % of 88) had a paired convalescent-phase urine sample collected. Of the 88 dengue cases, 53 (60.2 %) were confirmed by serum RT-qPCR (some also had a positive result in the DENV NS1 or IgM ELISA), 7 (8.0 %) by NS1-ELISA (some also had a positive result in the DENV IgM ELISA), and 28 (31.8 %) were positive solely by IgM ELISA seroconversion. The median age was 20, and 47 (53.4 %) were female. The frequency of primary and secondary infections, the infecting DENV serotypes, and the median RT-qPCR Cycle Threshold (CT) levels are shown in Table 1.

**Table 1:** Clinical, demographic and laboratory characteristics of patients with dengue at study enrollment, according to the primary method of diagnosis using serum.

Characteristics	Patients with laboratory evidence of dengue, according to the diagnostic method using serum^a^		
	Positive on RT-qPCR (n = 53)	DENV IgM seroconversion (n = 28)	Positive on DENV NS1 (n = 7)
	n (%) or median (interquartile range)		
Demography			
Female sex	28 (52)	15 (53)	4 (57)
Age	21 (12‒31)	19 (13‒35)	20 (10‒30)
Clinical manifestations			
Days post symptoms onset	3 (1‒3)	2 (1‒4)	4 (1‒7)
Fever	53 (100)	27 (96)	7 (100)
Headache	49 (92)	22 (79)	7 (100)
Myalgia	40 (75)	17 (61)	7 (100)
Arthralgia	33 (62)	13 (46)	5 (71)
Retro-orbital pain	36 (67)	13 (46)	7 (100)
Conjunctival hyperemia	25 (47)	9 (32)	6 (85)
Rash	14 (26)	4 (14)	3 (42)
Pruritus	17 (32)	5 (18)	4 (57)
Swollen joints	7 (13)	2 (7)	4 (57)
Abdominal pain	21 (39)	13 (46)	5 (71)
Cough	12 (22)	12 (43)	2 (29)
Sore throat	16 (30)	15 (54)	2 (29)
Laboratory results in serum			
RT-qPCR in acute-phase serum^b^			
Positive	53 (100)	0 (0)	0 (0)
Cycle threshold	23.9 (20.8‒30.1)	‒	‒
ELISA			
DENV NS1 antigen reagent	29 (55)	0 (0)	7 (100)
DENV IgM in acute sample	20 (38)	0 (0)	2 (29)
DENV IgM seroconversion^b^^,^^c^	22 (92)	28 (100)	1 (50)
DENV IgG in acute sample^d^	38 (72)	24 (86)	2 (33)
DENV type			
DENV-1	32 (60)	‒	‒
DENV-2	9 (17)	‒	‒
Undetermined	12 (23)	‒	‒
Laboratory results in urine			
Acute-phase urine^b^			
Positive	4 (8)	0 (0)	1 (14)
Cycle threshold	36.1 (34.7‒36.9)	‒	33.3
Days post symptoms onset	3 (1‒5)	‒	7 (7‒7)
Convalescent-phase urine^b^^,^^e^			
Positive f	2 (7)	0 (0)	0 (0)
Cycle threshold	35.0 (34.2‒35.8)	‒	‒
Days post symptoms onset	12 (11‒13)	‒	‒
Laboratory results in acute-phase oral fluid^b^			
Positive	1 (2)	0 (0)	0 (0)
Cycle threshold^g^	29.3 (29.3‒29.3)	‒	‒
Days post symptoms onset	1 (1‒1)	‒	‒

a Dengue patients confirmed by RT-qPCR in serum could also have DENV NS1 antigen or DENV IgM antibody detectable. Dengue patients confirmed by DENV NS1 antigen detection in serum could also have DENV IgM antibody detectable. In contrast, patients with dengue confirmed by DENV IgM seroconversion were negative by DENV RT-qPCR and DENV NS1 antigen.

b Acute- and convalescent-phase samples were obtained within 0‒7 post symptoms onset and 10‒45 days after collection of the acute-phase samples, respectively.

c Convalescent-phase sera were available and tested by DENV IgM ELISA for the following patients with a negative DENV IgM ELISA in the acute-phase sample: 24 qRT-qPCR-positive patients, 28 patients with DENV-IgM seroconversion, and 2 DENV-NS1-positive patients.

d Patients with DENV IgG antibodies detected in the acute-phase sample likely had a secondary symptomatic DENV infection. Conversely, those without DENV IgG antibodies likely had a primary symptomatic DENV infection. One patient confirmed only by DENV NS1 detection was not tested for the presence of DENV IgG antibodies.

e Convalescent-phase urine was available and tested by RT-qPCR for 53 patients (28 serum RT-qPCR-positive patients, 21 patients with DENV-IgM seroconversion, and 4 DENV-NS1-positive patients).

f The two patients with positive DENV RT-qPCR in the convalescent-phase urine sample were negative in the RT-qPCR performed in the acute-phase urine but were positive by RT-qPCR and NS1 ELISA performed in the acute-phase serum and also had DENV IgM seroconversion.

g The RT-qPCR cycle threshold for this patient's serum sample was 28.1.

Of the 88 cases, 5 (5.7 %) were positive for DENV by RT-qPCR in acute-phase urine, 4 of which initially tested positive on serum RT-qPCR and one who tested positive on DENV NS1 (Table 1). Considering only the 53 cases confirmed by serum RT-qPCR, the frequency in which acute-phase urine was positive was 7.5 % (4 cases).

Among 53 patients with convalescent-phase urine samples available, 2 (3.8 %) tested positive. They were also positive in RT-qPCR performed in the acute-phase serum but negative in the RT-qPCR performed in the acute-phase urine. These two samples were collected 11 and 13 days after symptom initiation. Considering only the 28 cases with convalescent-phase urine samples available and a positive serum RT-qPCR result, the frequency of positive convalescent-phase urine was 7.1 % (2 cases). Thus, of the 53 patients with a serum RT-qPCR positive result, 6 11.3 %) had detectable DENV RNA in a urine sample (4/53 in the acute-phase urine and 2/28 in the convalescent-phase urine.

Overall, median CTs in urine were higher than in sera (Table 1). However, serum CTs were lower among the 7 cases with a positive RT-qPCR in any urine sample compared to the 47 cases with negative urine but a positive RT-qPCR in the serum (Table 2). The urine-positive group also had fewer secondary infections, yet the limited sample precludes a definitive conclusion on these differences.

**Table 2:** Laboratory characteristics of the 54 dengue patients who tested positive for DENV by RT-qPCR, according to the RT-qPCR result in the urine samples.

Laboratory characteristics regarding the serum samples	Positive urine in RT-qPCR^a^ (n = 7)	Negative urine in RT-qPCR (n = 47)^b^
	n (%) or Median (Interquartile range)	
Days between symptom onset and sample collection	3 (1‒6)	3 (1‒3)
RT-qPCR in acute-phase serum		
Positive c	6 (86)	47 (100)
Cycle threshold	21.8 (15.03‒37.33)	23.94 (20.28‒29.71)
ELISA		
DENV IgG in acute sample	4 (57)	35 (74)
DENV type c		
DENV-1	2 (33)	10 (21)
DENV-2	2 (33)	30 (64)
Undetermined	2 (33)	7 (15)

a Five out of the seven patients had positive RT-qPCR for DENV on acute-phase and two on convalescent-phase urine samples.

b The 47 dengue patients with a negative urine RT-qPCR for DENV had a positive serum RT-qPCR for DENV.

c One of the patients who tested positive for DENV via urine RT-qPCR had an initial diagnosis based solely on a positive DENV NS1 ELISA, and therefore, DENV typing was not attempted.

One patient tested positive by RT-qPCR in acute-phase oral fluid (1.1 % of 88 cases and 1.9 % of the 53 patients with RT-qPCR-positive serum) with a CT of 29 but was not RT-qPCR-positive in the urine.

The overall detection rate of DENV RNA in urine or oral fluids (9.1 %) was much lower than in serum (60.2 %). In addition, the median CTs for the qRT-PCR positive sera were lower than those for urine and oral fluid (Table 1). Considering only the analysis of the urine samples, we found that 7 (8.0 %) of the 88 dengue-confirmed cases had urine samples positive for DENV RNA by RT-qPCR. Among those confirmed by serum RT-qPCR, the frequency of RNA detection in urine was similar between the acute (7.5 % of 53) and convalescent (7.1 % of 28) phases. Yet, the low frequency in which we found positive urine samples indicates that further investigation is needed to determine whether surveillance of human wastewater for dengue could be useful.

In contrast to our findings, a prior investigation showed that DENV RNA in urine can be found in > 50 % of the dengue cases after the acute phase of illness, when detection in the serum was < 50 %, suggesting continued urine shedding of DENV RNA after viremia and the appearance of antibodies.^9^ Another study also identified delayed excretion of the DENV RNA in urine compared to serum.^10^ Although we did not compare the presence of DENV RNA between convalescent-phase sera and urine, our finding of equivalent frequencies of DENV RNA detection in acute- and convalescent-phase urine may be explained by an intermittent or delayed excretion of the virus in urine.

A limitation of our study was that we did not quantify the DENV viral load in the biological specimens, nor investigate whether the DENV RNA excreted in urine would be detectable (and for how long) after being diluted in wastewater. We also did not explore whether feces could serve as a source of DENV RNA shedding into wastewater. However, DENV RNA was detected in wastewater in the city of Miami, US, despite a low level of clinical case detection (with a weekly incidence estimated at 0.77‒4.23 cases per 1,000,000 people), suggesting the feasibility of employing WBE for DENV surveillance.^5^

While WBE may prove to be a valuable tool for monitoring DENV transmission and forecasting epidemics, more work is needed to understand whether DENV RNA is shed in feces, determine differences in DENV RNA shedding between symptomatic and asymptomatic infections, estimate how long DENV RNA remains detectable in wastewater before degradation, and assess whether increased sensitivity assays, such as digital PCR, can improve DENV RNA detection. Understanding these critical parameters that influence viral detection in wastewater will help determine the potential for dengue surveillance on human wastewater.

## Ethics approval

The study was conducted in accordance with the Declaration of Helsinki and approved by the Ethics Committee of Gonçalo Moniz Institute, Oswaldo Cruz Foundation, Salvador, Brazil (CAAE: 55,904,616.4.0000.0040, n° 1.642.535). Informed consent was obtained from all subjects involved in the study.

## Disclosure

This work was solely prepared by the authors. During the review process, the authors used Grammarly and ChatGPT only to improve the language and readability of the manuscript. After using these tools, the authors reviewed and edited the content as needed. The authors take full responsibility for the final content of this publication.

## Conflicts of interest

The authors declare no conflict of interest. The funders had no role in the design of the study; in the collection, analyses, or interpretation of data; in the writing of the manuscript, or in the decision to publish the results.
